# Nephrotic Syndrome Thromboprophylaxis With Direct Oral Anticoagulants or Vitamin K Antagonists

**DOI:** 10.1016/j.ekir.2025.02.028

**Published:** 2025-03-03

**Authors:** Edouard Cubilier, Youcef Chergui, Cyril Garrouste, Ines Ramos, Carole Philipponnet, Alba Atenza, Clarisse Greze, Julien Aniort, Charlotte Uro-Coste, Anne-Elisabeth Heng

**Affiliations:** 1 Nephrology Department, Clermont-Ferrand University Hospital, Clermont-Ferrand, France; 2 Biostatistics Unit, DRCI, Clermont-Ferrand University Hospital, Clermont-Ferrand, France

**Keywords:** direct oral anticoagulant, nephrotic syndrome, thromboprophylaxis, vitamin K antagonist

## Abstract

**Introduction:**

Nephrotic syndrome (NS) is a pathological state of the glomerular filtration barrier associated with an increased venous and arterial thrombotic risk. Current guidelines suggest heparin-based or vitamin K antagonist (VKA) regimens for thromboprophylaxis in such patients. Although widely prescribed for other indications, direct oral anticoagulants (DOACs) are not recommended in NS because of limited pharmacological and safety reports. This study aimed to compare DOACs and VKAs for thromboprophylaxis in NS, specifically regarding thrombotic events (TEs) and bleeding events (BEs).

**Methods:**

We conducted a retrospective monocentric analysis of recorded NS episodes that required prophylactic anticoagulation between January 2006 and December 2023. We included 133 NS episodes of which 51 were treated with DOACs and 82 with VKAs. The primary endpoint was a composite endpoint, including thrombosis occurrence and major or clinically significant BEs during thromboprophylaxis. The secondary endpoints consisted of relevant features potentially involved when each primary endpoint was considered independently.

**Results:**

Patient characteristics, underlying NS etiology, personal thrombotic and bleeding risk factors, and biological parameters were globally similar in both groups. The primary endpoint appeared similar in both groups (*P* = 0.481). The secondary endpoints were mostly hypothesis-generating because of the low TE (*n* = 2) and BE (*n* = 7) occurrences.

**Conclusion:**

This study provides reassuring clinical data on DOAC use in NS thromboprophylaxis compared with VKAs, the recommended therapy, and calls for confirmation in randomized controlled trials (RCTs) and larger pharmacological studies.

## Graphical abstract

**Figure:**
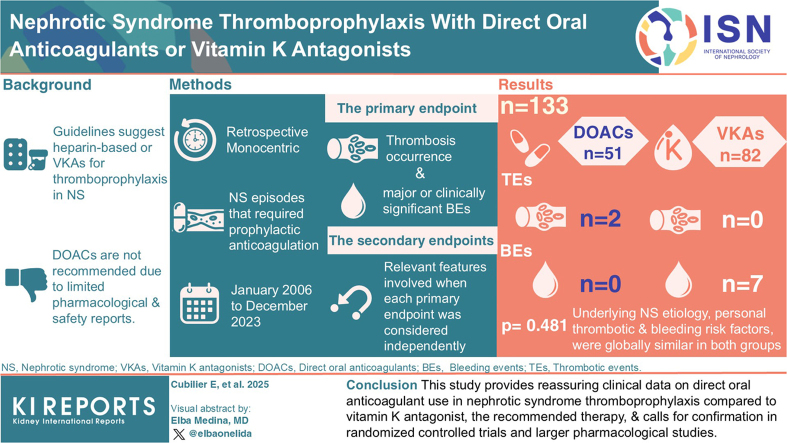

NS is a pathological state of glomerular permeability resulting in plasma protein loss in urine, defined by hypoalbuminemia < 30 g/l and proteinuria > 3.5 g/d (or > 3 g/g of creatinine).^1^ Etiologies responsible for NS may be primarily glomerular (minimal change disease, focal segmental glomerulosclerosis, or membranous nephropathy [MN]), or secondary to systemic diseases (i.e., diabetes, lupus, amyloidosis).1 NS often favors edema, infections, hypertension, hyperlipidemia, and thromboembolism.^1^

During NS, thromboembolism appears as one of the most critical complications.^2^ It may involve deep and distal veins and arteries, with incidence rates reported in the literature ranging from 2% to 37%.^3^ Thrombosis generation in NS is favored by multiple factors, such as older age, personal comorbidities, genetic predisposition for hypercoagulability, intravascular volume depletion status, hyperlipidemia, the underlying glomerular disease type, the 6-months-period following NS diagnosis,^2^ hypoalbuminemia depth, and proteinuria severity.^3^, ^4^, ^5^

Several imbalances involved during NS seem to generate a hypercoagulable climate. Impaired components of the glomerular filtration barrier (glomerular endothelial cells, basal membrane, and podocyte foot processes) allow negatively charged blood proteins of the size of albumin (approximately 66 kDa) or greater depending on damage intensity, to leak into the urinary chamber.^2^ Consequently, serum concentrations of endogenous coagulation regulators antithrombin III (65 kDa) and free protein S (69 kDa) may decrease,^2^^,^^6^, ^7^, ^8^ hindering protein C activity.^6^^,^^9^ Low albumin levels trigger hepatocyte acute-phase procoagulant, fibrinogen, and antifibrinolytic protein expression.^6^ NS conditions also promote platelet hyperactivity,^5^^,^^6^ a tighter fibrin meshwork resistant to fibrinolysis within newly formed thrombi,^2^^,^^5^ and hinders fibrinolysis.^2^^,^^6^

Current practice on prophylactic anticoagulation suggests an individualized approach considering hypoalbuminemia severity, the underlying glomerular disease responsible for NS, thrombotic risk factors, and the patient’s thrombotic and bleeding risk scores before treatment.^1^^,^^8^ Previous reports describe MN as a highly thrombogenic etiology in NS,^1^^,^^8^^,^^10^, ^11^, ^12^ whereas fewer TEs are associated with diabetic nephropathy.^12^ Although the benefits of prophylactic anticoagulation in patients with NS have yet to be demonstrated in large RCTs,^1^ available studies show a reduced risk of thrombotic episodes in patients with NS with low-molecular-weight heparin or warfarin thromboprophylaxis compared with untreated patients.^13^ Therefore, the Kidney Disease: Improving Global Outcomes Glomerular Diseases Work Group (2021) recommends either heparin, low-molecular-weight heparin, or warfarin while the NS persists.^1^^,^^8^

Heparin, like low-molecular-weight heparin, has the disadvantage of requiring daily injections and targeting antithrombin III which may be leaking in urine during NS.^5^ Low-molecular-weight heparin also accumulates in the context of renal failure with creatinine clearance < 30 ml/min or 20 ml/min for tinzaparin.^7^^,^^8^ Vitamin K antagonists such as warfarin, are the most studied anticoagulants in NS.^14^ They may be used regardless of kidney function because their elimination is extrarenal^15^; however, they have the disadvantages of being highly protein-bound with delayed efficacy, prolonged activity after suspension, and requiring frequent dose adjustments to easily fluctuating international normalized ratios (INRs).^8^^,^^15^

Direct oral anticoagulants (DOACs) such as apixaban and rivaroxaban, are factor Xa inhibitors commonly used for thromboprophylaxis in other clinical settings such as atrial fibrillation.^11^^,^^15^ These drugs are easy to administer, do not require blood monitoring, have limited renal clearance, and have fewer drug interactions than warfarin,^15^ but are also highly bound to protein.^14^^,^^16^ They currently are not recommended for NS thromboprophylaxis, because of a lack of data on safety, pharmacodynamics (PD), and pharmacokinetics (PK).^1^^,^^8^^,^^14^^,^^16^

The available studies on DOAC efficacy or bleeding adverse outcomes in NS thromboprophylaxis are scarce and of small sample sizes, but seem reassuring.^11^^,^^12^^,^^17^, ^18^, ^19^ A previous retrospective comparative study between DOACs and warfarin showed similar bleeding and thrombotic episodes in patients with NS.^19^ To obtain safety and efficacy data on DOACs in NS thromboprophylaxis, we compared bleeding (BEs) and thrombotic events (TEs) during DOAC or VKA anticoagulation.

## Methods

### Population and General Data

**Figure 1:**
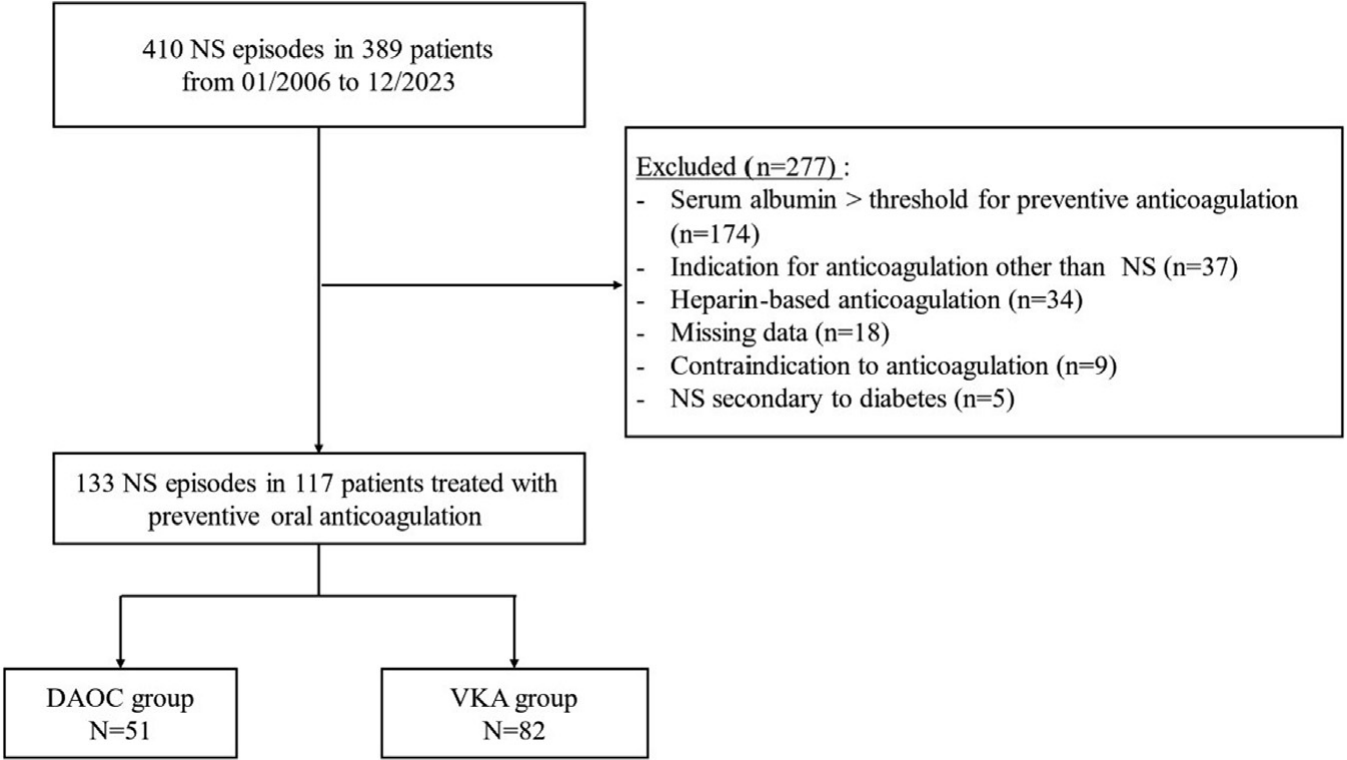
Flow chart. DOAC, direct oral anticoagulant; NS, nephrotic syndrome; VKA, vitamin K antagonist.

We conducted a retrospective single-center study on adult patients treated in the nephrology department of Clermont-Ferrand University Hospital, France. We included NS episodes recorded from January 2006 to December 2023 with an indication for preventive anticoagulation, that is, albuminemia < 20 g/l or 25 g/l in case of MN,^1^ treated with either VKAs or DOACs depending on the clinician’s initiative. Indications for anticoagulation other than NS were excluded, as well as underlying diabetic nephropathy for which thromboprophylaxis benefits remain undetermined in the literature (Figure 1).^12^

The following patient demographical, clinical, and biological data were collected from their medical files: age, sex (reported in this study as male or female biological attributes associated with physical and physiological features including chromosomes, gene expression, hormone function and reproductive/sexual anatomy), personal thrombotic risk factors, elements required for the ATRIA^20^ and HAS-BLED scores,^21^ biological parameters, renal histopathology, NS immunosuppressive therapies received, and anticoagulation treatment duration. The Framingham risk score,^1^^,^^3^ although recommended by the 2021 kidney disease: improving global outcomes guidelines^1^ to predict arterial thrombosis in NS, could not be used in this study because of frequently unreferenced data required for its use, particularly serum cholesterol levels.

This study was approved by the local Ethics committee (IRB 00013412, “CHU de Clermont-Ferrand IRB #1”, IRB number 2024-CF318) according to the French policy on personal data protection.

### Group Definition

NS episodes were organized into 2 groups depending on the type of anticoagulation received. A total of 51 NS episodes were treated with DOACs, mostly apixaban (*n* = 46, 90%), followed by dabigatran (*n* = 3, 6%), and rivaroxaban (*n* = 2, 4%), depending on the clinician’s initiative. The patients received either a full or adjusted dose according to the respective prescription recommendations.

The other group gathered 82 NS episodes treated with VKAs with a targeted INR range between 2 and 3, mostly comprising fluindione (*n* = 45, 55%), followed by warfarin (*n* = 26, 32%), and acenocoumarol (*n* = 11, 13%).

### End Points and Event Definition

The primary endpoint was a composite endpoint, including thrombosis occurrence and major or clinically significant BEs during thromboprophylaxis. BEs and TEs were identified from patient records.

Major bleeding was defined as either fatal, located in a life-threatening and functionally impairing location (i.e., cranial, medullar, ocular, pericardial, retroperitoneal, articular, muscular with compartment syndrome), responsible for a hemoglobin level drop > 2 g/d, or requiring 2 or more transfusions of whole blood or red blood cell units.^22^ Clinically significant bleeding is characterized by unmet major bleeding criteria in which nonsurgical medical interventions, hospitalizations, or rapid clinical evaluations were required.^23^

The secondary endpoints consisted of relevant features potentially involved in each primary endpoint (i.e., the occurrence of thrombosis, major bleeding, and clinically significant bleeding) when considered independently.

### Statistical Analyses

Statistical analyses were performed using R software (version 4.3.1, R Core Team, Vienna, Austria). All tests were 2-sided, with a type-I error level set at 5%. Categorical data were described as numbers and associated percentages, and continuous data as means ± SD or as medians (first and third quartiles). For categorical variables, characteristic data and thrombosis or bleeding occurrence were compared between the 2 groups of anticoagulant treatment using the chi-square test, or the Fisher exact test if the conditions of the chi-square test were not respected and with a Mann-Whitney test for the continuous variables. A Kaplan-Meier curve was used to represent the risk of bleeding or thrombosis depending on the treatment category and exposure duration. A log-rank test was then performed to compare the event-free survival between the 2 treatment groups.

## Results

### Patient Characteristics

Patient characteristics at baseline of anticoagulation are displayed in Table 1. At the time of treatment, the mean age was 51.3 ± 19.7 years and male sex was predominant (69.2%). Risk factors for thrombosis were similar between both groups. Bleeding risk factors appeared significantly more prevalent in the VKA group, with a mean ATRIA score of 2.4 ± 2.4 compared with 1.5 ± 2.0 in the DOAC group (*P* = 0.021), and a mean HAS-BLED score of 1.2 ± 1.2 compared with 0.7 ± 0.9 in the DOAC group (*P* = 0.017). The biological parameters were similar between groups with a mean proteinuria of 10.3 ± 7.7 g/24 h or g/g of creatinine at the time of treatment, a mean serum albumin of 14.2 ± 4.0 g/l, and an estimated glomerular filtration rate (eGFR) of 69.7 ± 32 ml/min per 1.73 m^2^. The most prevalent etiology of NS was minimal change disease with an occurrence of 30.8% (22% in the VKA group and 45.1% in the DOAC group, *P* = 0.005), followed by 29.3% of MN (31.7% in the VKA group and 25.5% in the DOAC group, *P* = 0.444) and 16.5% of focal segmental glomerulosclerosis (18.3% in the VKA group and 13.7% in the DOAC group, *P* = 0.49).

**Table 1:** Baseline characteristics of the included nephrotic syndrome episodes treated with direct oral anticoagulants or vitamin K antagonists

Characteristics	Overall *N* = 133	VKA *n* = 82	DOAC *n* = 51	*P* value^a^
Male sex, *n* (%)	92 (69.2%)	54 (65.9%)	38 (74.5%)	0.293
Age at time of treatment (yr)	51.3 ±19.7	53.5 ±19.4	47.8 ±19.9	0.127
Risk factors of VTE				
History of VTE, *n* (%)	6 (4.5%)	2 (2.4%)	4 (7.8%)	0.203
Smoking, *n* (%)	26 (19.5%)	16 (19.5%)	10 (19.6%)	0.989
BMI > 30 kg/m^2^, *n* (%)	9 (6.8%)	6 (7.3%)	3 (5.9%)	>0.999
Oral contraceptive, *n* (%)	2 (1.5%)	1 (1.2%)	1 (2.0%)	>0.999
History of cancer, *n* (%)	15 (11.3%)	11 (13.4%)	4 (7.8%)	0.323
ATRIA score	2.0 ±2.3	2.4 ±2.4	1.5 ±2.0	0.021
HAS-BLED score	1.0 ±1.1	1.2 ±1.2	0.7 ±0.9	0.017
Biological parameters				
Proteinuria (g/24h or g/g creatinine)	10.3 ±7.7	9.9 ±6.4	10.9 ±9.4	0.965
Serum albumin (g/l)	14.2 ±4.0	14.1 ±3.8	14.4 ±4.3	0.795
eGFR (ml/min per 1.73 m^2^)	69.7 ±32.0	66.7 ±33.6	74.5 ±28.8	0.244
Histopathology				0.105
Minimal change disease, *n* (%)	41 (30.8%)	18 (22.0%)	23 (45.1%)	0.005
Membranous nephropathy, *n* (%)	39 (29.3%)	26 (31.7%)	13 (25.5%)	0.444
Focal segmental glomerulosclerosis, *n* (%)	22 (16.5%)	15 (18.3%)	7 (13.7%)	0.491
Amyloidosis, *n* (%)	18 (13.5%)	13 (15.9%)	5 (9.8%)	0. 321
Membranoproliferative glomerulonephritis, *n* (%)	10 (7.5%)	8 (9.8%)	2 (3.9%)	0.316
Lupus nephritis, *n* (%)	3 (2.3%)	2 (2.4%)	1 (2.0%)	>0.999

ATRIA score, anticoagulation and risk factors in atrial fibrillation score; BMI, body mass index; DOAC, direct oral anticoagulant; eGFR: estimated glomerular filtration rate; NS, nephrotic syndrome; VKA, vitamin K antagonist; VTE, venous thromboembolism..

Data are presented as the number of patients (percentages), mean ± SD, or median (25th; 75th percentiles).

a Fisher exact test.

### Occurrence of Events

**Figure 2:**
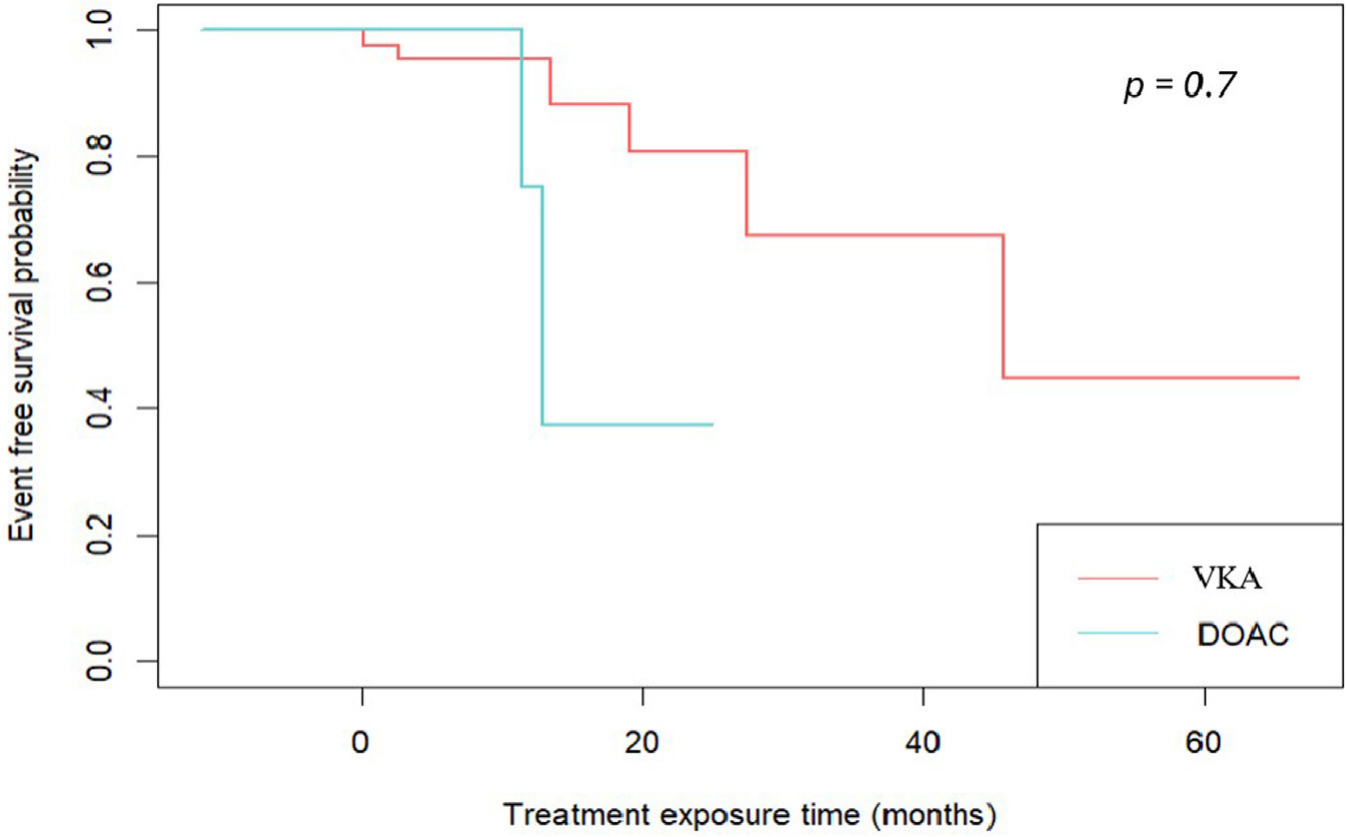
Kaplan-Meier curve comparative representation of the event-free survival during nephrotic syndrome thromboprophylaxis with direct oral anticoagulants or vitamin K antagonists. *P* value obtained from a log-rank test between groups. DOAC, direct oral anticoagulant; VKA, vitamin K antagonist.

The primary endpoint (Table 2) was observed in 9 cases (6.8%) with similar occurrence rates (*P* = 0.481) between the VKA (8.5%) and the DOAC group (3.9%). BEs only occurred in patients from the VKA group (8.4%, *P* = 0.045), with 4 clinically significant bleedings (4.9%) and 3 major bleedings (3.7%). Corticosteroids were used in 69.9% of NS episodes (67.1% in the VKA group vs. 74.5% in the DOAC group, *P* = 0.363). Rituximab was significantly more used (*P* < 0.001) in the DOAC group (68.6%) than in the VKA group (36.6%). Overall, mean anticoagulation time was 2.9 (0.9; 6.7) months, with a significantly longer median exposure of 3.4 (1.3; 8.8) months in the VKA group compared with 1.6 (0.7; 4.2) months in the DOAC group (*P* = 0.011), as represented in Figure 2. None of the BEs or TEs were fatal, and there was no event-free survival difference between DOAC and VKA administration during NS (Figure 2).

**Table 2:** Primary endpoint occurrence during direct oral anticoagulant or VKA thromboprophylaxis in nephrotic syndrome, associated immunosuppressive therapies, and anticoagulation time

Primary endpoint features and associated therapies	Overall *N* = 133	VKA *n* = 82	DOAC *n* = 51	*P* value*^1^*
Primary endpoint, *n* (%)	9 (6.8%)	7 (8.5%)	2 (3.9%)	0.481
Thrombosis, *n* (%)	2 (1.5%)	0 (0.0%)	2 (3.9%)	0.145
Bleeding, *n* (%)	7 (5.3%)	7 (8.4%)	0 (0.0%)	0.045
Clinically significant bleeding, *n* (%)	4 (3.0%)	4 (4.9%)	0 (0.0%)	0.298
Major bleeding, *n* (%)	3 (2.3%)	3 (3.7%)	0 (0.0%)	0.285
Associated NS treatment				
Corticosteroids, *n* (%)	93 (69.9%)	55 (67.1%)	38 (74.5%)	0.363
Cyclophosphamide, *n* (%)	18 (13.5%)	12 (14.6%)	6 (11.8%)	0.638
Rituximab, *n* (%)	65 (48.9%)	30 (36.6%)	35 (68.6%)	<0.001
Ciclosporin, *n* (%)	18 (13.5%)	12 (14.6%)	6 (11.8%)	0.638
Anticoagulant time of treatment (mo)	2.9 [0.9, 6.7]	3.4 [1.3, 8.8]	1.6 [0.7, 4.2]	0.011

DOAC, direct oral anticoagulant; NS, nephrotic syndrome; VKA, vitamin K antagonist.

Data are presented as the number of episodes (percentages).

*P* values were calculated from Fisher exact test.

The details from each TE (cases 1 and 2) or BE (cases 3–9) are displayed in Table 3. Regarding thrombosis, TEs only occurred in apixaban-treated patients from the DOAC group (3.9%, *P* = 0.145). The 62-year-old male in case 1 who had a history of cancer, presented a left ventricular thrombosis 1 year after amyloidosis-related NS was diagnosed, in the context of severe heart failure, 13 g/l albuminemia, 4 g/g proteinuria, and a reduced eGFR of 20 ml/min per 1.73 m^2^ (Table 3). In case 2, incidental renal thrombosis and pulmonary embolism were found during a workup computed tomography scan 1 month after MN-related NS was diagnosed. At the time of thrombosis discovery, the patient in case 2 was a 54-year-old male smoker who had 14 g/l albuminemia, 11 g/g proteinuria, and a normal eGFR of 95 ml/min per 1.73 m² (Table 3).

**Table 3:** Features of the thrombotic or bleeding events in nephrotic syndrome episodes treated with prophylactic direct oral anticoagulants or vitamin K antagonists

Features	Case 1	Case 2	Case 3	Case 4	Case 5	Case 6	Case 7	Case 8	Case 9
	Thrombotic event		Bleeding event						
			CS	CS	Major	Major	Major	CS	CS
Age (yr)	62	54	44	59	61	22	47	51	64
Sex (F/M)	M	M	F	M	M	F	M	M	M
VTE history	No	No	/	/	/	/	/	/	/
Smoking	No	Yes	/	/	/	/	/	/	/
BMI > 30 kg/m^2^	No	No	/	/	/	/	/	/	/
Cancer history	Yes	No	/	/	/	/	/	/	/
ATRIA score	/	/	0	1	1	3	0	2	1
HAS-BLED score	/	/	0	3	2	0	0	0	3
Albuminemia at NS diagnosis / event (g/l)	16 / 13	14 / 14	16 / 16	17 / 20	10 / 17	8 / 8	8 / 12	20 / 20	14 / 14
Proteinuria at NS diagnosis / event (g/24 h or g/g creatininuria	10 / 4	9 / 11	5 / 5	4 / 8	4.5 / 10	10 / 10	11 / 10	6 / 4	6 / 6
eGFR at NS diagnosis/event (ml/min per 1.73 m^2^)	55 / 20	65 / 95	120 / 120	100 / 118	80 / 95	120 / 70	120 / 26	56 / 55	50 / 50
Underlying histopathology	AL	MN	FSGS	MN	MN	FSGS	MN	FSGS	AL
Treatment time to event	1 yr	1 mo	5 d	13 mo	2 yr	19 mo	3 yr	3 mo	6 d
Treatment type	Apix	Apix	Fluin	Fluin	Fluin	Fluin	Fluin	Fluin	Fluin
INR at bleeding time	/	/	NF	NF	NF	NF	6.4	NF	2.3

Event details and context									

Case 1	Left ventricular thrombosis discovered in the context of severe heart failure 1 year after amyloidosis-related NS diagnosis								
Case 2	Incidental renal thrombosis and pulmonary embolism discovery on workup CT scan 1 month after MN-related NS diagnosis								
Case 3	Perirenal hematoma secondary to a renal biopsy with 1 g/dl hemoglobin serum level reduction								
Case 4	Rectorrhagia requiring treatment by rectal endoscopy								
Case 5	5 g/dl hemoglobin serum level reduction requiring transfusions of 5 RBC units, and no imputable etiology found during the assessment								
Case 6	Severe metrorrhagia requiring transfusion of 2 RBC units								
Case 7	Gastro-intestinal tract hemorrhage requiring transfusion of 5 RBC units								
Case 8	Spontaneous intramuscular calf hematoma with 2 g/dl hemoglobin serum level reduction								
Case 9	Gastrointestinal tract hemorrhage requiring treatment by endoscopy								

AL, amyloidosis; Apix, apixaban; BMI, body mass index; CS, clinically significant; CT, computed tomography; DOAC: direct oral anticoagulant; eGFR: estimated glomerular filtration rate; Fluin, fluindione; FSGS, focal and segmental glomerulosclerosis; INR, international normalized ratio; MN, membranous nephropathy; NF, not found; NS, nephrotic syndrome; RBC, red blood cells; VKA, vitamin K antagonist; VTE, venous thromboembolism.

The BEs (cases 3–9) only occurred in the VKA group, with a significant statistical difference (*P* = 0.045) compared with the DOAC group (Table 2). The average ATRIA predictive bleeding score was 1.9 ± 2.2 and 1.0 ± 1.1 for the HAS-BLED score, similar to those of our overall population (2.0 ± 2.3 and 1.0 ± 2.1, respectively). BEs only occurred in fluindione-treated patients within 5 days to 3 years after time of treatment with a median of 12.8 (0.9; 22.7) months. The average anticoagulation time frame was significantly longer in the VKA group than in our global population. In case 7, bleeding was related to an INR of 6.4. In 5 out of 7 cases, the INR at the time of the event was not measured or mentioned in the patient’s file. Clinically significant BEs (cases 3, 4, 8, and 9) occurred within 14 to 20 g/l hypoalbuminemia, 4 to 8 g/g proteinuria, eGFR > 30 ml/min per 1.73 m^2^, and 5 days to 13 months from VKA initiation. Major bleeding (cases 5, 6, and 7) appeared within 8 to 17 g/l hypoalbuminemia, 10 g/g proteinuria, < 30 ml/min per 1.73 m² eGFR in case 7, and from 19 months to 3 years after treatment initiation.

## Discussion

Our retrospective study represents one of the largest series of NS episodes anticoagulated with DOACs yet published, providing critical input in this particular clinical frame. During NS, protein loss in urine is believed to increase the DOAC fraction unbounded to protein, thus raising safety concerns because their PK and PD have scarcely been studied in this specific context.^13^ In a recent single-institution parallel-arm phase 1a study (NCT02599532), safety and PK-PD of a single 10 mg apixaban dose during NS (*n* = 8) compared with healthy controls (*n* = 11) on a 48-hour observation period was reported safe to use. Similar PK and PD were found in patients with NS and controls, except for lower total apixaban area under the curve and greater protein-unbound-apixaban fractions in severe NS, which could have clinical implications in a steady-state administration regimen with larger sample sizes.^14^ A retrospective analysis of 27 patients with NS treated with apixaban also showed anti-Xa apixaban blood levels comparable with those of patients without NS from large clinical trials, with only 1 TE and no minor or major BE reported.^13^ These data suggest a safe use of apixaban in NS and call for extended PK-PD tests in larger samples.

TEs in our study (Table 3) occurred in 2 distinct patients (cases 1 and 2) treated with apixaban in the DOAC group and none in the VKA group, without reaching statistical significance (*P* = 0.145). Both patients had known thrombotic risk factors (smoking or a history of cancer), and NS thrombotic risk factors (adult age,^2^ severe hypoalbuminemia, heavy proteinuria).^2^^,^^3^ MN, the most thrombogenic glomerular disease in NS,^1^^,^^8^^,^^11^^,^^12^ was present in case 2. TE appeared within the first 6 months after its diagnosis, during the highest thrombotic risk period known in adults.^2^^,^^5^ This incidental finding during an MN-related workup computed tomography scan may have already been present at the time of NS diagnosis rather than developed despite anticoagulation, because patients bearing renal vein thrombosis and pulmonary embolism during NS may be asymptomatic.^5^ Because of this characteristic and the retrospective nature of this study, several undetected TEs could be unveiled in RCTs designed with systematic initial imageries for the included patients.

In the context of worsening kidney function and severe heart failure, a left ventricle thrombosis was found 1 year after amyloidosis-related NS was diagnosed in case 1 (Table 3). Apixaban, the most studied DOAC in chronic kidney disease stages 4 and 5, has been reported as safe and efficient for stroke prevention during atrial fibrillation at adjusted dose regimens with creatinine clearance beneath 30 ml/min.^15^ Although for a different indication, these data suggest that the TE in case 1 is probably more attributable to severe heart failure than apixaban inefficacy when kidney function is altered. In a retrospective single-center study on patients with NS (*n* = 21) treated with DOACs (apixaban, *n* = 10; rivaroxaban, *n* = 11), Kelddal *et al.* reported no TE or recurrence,^18^ which concords with the tendency in our results. Moreover, Tijani *et al.* found no significant thrombotic risk difference in a retrospective cohort study comparing adult patients with NS (*n* = 44) treated with DOACs (*n* = 25) or warfarin (*n* = 19), with only 1 new TE episode in a patient receiving apixaban 2.5 mg twice daily.^19^ These data collectively suggest that DOACs efficiently prevent TEs during NS, and would be a reasonable testing option in large RCTs for confirmation.

Our study gathered 4 clinically significant and 3 major BEs (Table 3, cases 3–9), all in patients from the VKA group and none from the DOAC group (Table 2, *P* = 0.045). In atrial fibrillation, it has been thoroughly demonstrated that DOACs for stroke prevention induce similar-to-lower bleeding rates compared with VKAs,^15^ as found in our data. Available studies in NS are more limited but similar to our results, thromboprophylaxis with warfarin has previously been associated with an increased major BE tendency compared with DOACs.^19^ Interestingly, all our reported BEs appeared with fluindione use, predominant in the VKA group (*n* = 45, 55%). Intraclass differences in the VKA subtype used for NS thromboprophylaxis could be associated with a greater bleeding incidence in this less-prescribed VKA than warfarin,^15^ granted that no literature supporting this hypothesis has been published.

Elevated predictive bleeding scores were only found in cases 4 and 9 (HAS-BLED = 3), whereas every other BE had low to moderate predictive scores. ATRIA and HAS-BLED scores were validated for atrial fibrillation, and the bleeding prediction tool provided by the 2021 Kidney Disease: Improving Global Outcomes guidelines was originally developed for MN^1^^,^^20^^,^^21^; therefore, they are probably not powered enough to predict bleeding in an undifferentiated NS context.^8^

In case 7, a major BE occurred 3 years after time of treatment with an INR of 6.4 and renal failure (eGFR < 30 ml/min per 1.73 m^2^) at bleeding time. This was probably favored because patients with chronic kidney disease are known to have reduced time in the therapeutic range, increased INR fluctuations, and increased bleeding risk while in supratherapeutic INRs compared to healthy controls.^15^

Among the events we recorded, the major bleedings stemmed in patients with prolonged VKA anticoagulation, the worst hypoalbuminemia (8 to 10 g/l in cases 6 and 7), and the heaviest proteinuria (10 g/g creatinine). Prolonged and severe hypoalbuminemia has been previously suggested as a risk factor for bleeding during NS,^12^ in which the bleeding risk increases under anticoagulation.^12^ The longer VKA exposures in our study (Table 2, *P* = 0.011) could reflect patient selection bias involving more severe underlying glomerular diseases and greater resistance to treatments in the VKA group.

The conclusions in our work bear several limitations worth noting. The primary endpoint’s statistical power is hindered by the study’s retrospective design, the relatively small sample size attributed to the rarity of the glomerulopathies and the related NSs, and the small number of total events reported. The secondary endpoints were mostly hypothesis-generating because of the low TE (*n* = 2) and BE (*n* =7) rates which limit robust statistical analysis, and prevent multivariate analysis which could address missing data such as unmentioned INR measurements during BEs in the VKA group.

## Conclusion

This study indicates that during NS, TEs and BEs with DOAC and VKA thromboprophylaxis are not significantly different when adjusted to time on anticoagulants. The few TEs and the absence of significant bleeding associated with DOACs in our work add much-needed clinical data to recent DOAC safety and PK-PD studies. This supports a reassuring use of DOACs in NS and calls for confirmation in RCTs and broader pharmacological studies on anticoagulants in this particular context.

## Disclosure

All the authors declared no competing interests.
